# Modeling phonotaxis in female *Gryllus bimaculatus* with artificial neural networks

**DOI:** 10.1186/1471-2202-12-S1-P234

**Published:** 2011-07-18

**Authors:** Gundula Meckenhäuser, Matthias R Hennig, Martin P Nawrot

**Affiliations:** 1 Freie Universität Berlin, Berlin, Germany; 2 Bernstein Center for Computational Neuroscience Berlin, Germany; 3 Behavioural Physiology Group, Department of Biology, Humboldt-Universität zu Berlin, Berlin, Germany

## 

**Figure 1:**
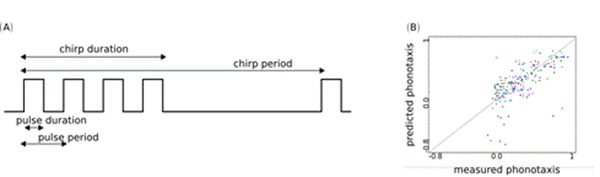
(A) Temporal features of courtship songs: pulse duration, pulse period, chirp duration, chirp period. (B) Correlation between the model's phonotaxis and the female's phonotaxis evaluated for five fixed neural networks with 3 hidden neurons: Each color represents a network and each dot corresponds to a song.

Courtship songs are used by female Gryllus bimaculatus to assess the quality of their potential mating partner. Typically, a song consists of chirps composed of few pulses followed by a chirp pause (Figure 1A). In extensive behavioral experiments, phonotactic responses of female crickets were tested by varying song features such as duration of pulse, pulse-pause, pulse-period, etc. [1]. Although several features of the song have been determined to affect the attractiveness of a song, the exact mechanisms underlying the female’s evaluation process are yet unclear.

Here, we present a feed-forward artificial neural network that quantitatively predicts the attractiveness of mating songs. Our approach was motivated by recent work from Wittmann et al. [2] who presented a network that analyzes and evaluates courtship songs of grasshoppers (*Chorthippus biguttulus*). We studied networks consisting of twelve neurons in the input layer, each one representing a feature of a given song, a variable number of n = 1, … , 15 neurons in the hidden layer and one output neuron that represents the phonotaxis. The neurons from one layer to the next are all-to-all connected via synaptic weights. The weights are trained with 160 artificial courtship songs for which the phonotaxis had already been determined in experiments. For training our networks, we used the backpropagation algorithm. We show that the mean squared error computed from a test set of 40 courtship songs is minimal for artificial neural networks with n = 3 hidden neurons. To estimate the predictive power of 3-hidden-neurons networks, we analyze the correlation between the model’s phonotaxis and the female’s phonotaxis. Figure 1B. shows phonotaxis values predicted by 3-hidden-neurons networks versus the values measured in experiments. Each color represents one fixed network. The best performing network yielded a mean squared error of 0.05*. *Thus, our model can be used for a quantitative prediction of the attractiveness of untested courtship songs and so it complements experimental testing of female phonotaxis in the laboratory.
